# Development and validation of a model of severe abdominal blast injury: characterization and hemostatic response

**DOI:** 10.3389/fvets.2026.1745092

**Published:** 2026-10-14

**Authors:** Guo-xing Huang, Jin-long Wang, Min Tian, Tong-yu Sun, Xin-lou Li, Yuan Yang, Meng Wang

**Affiliations:** 1 Department of Orthopedics, The Ninth Medical Center of PLA General Hospital, Beijing, China; 2 Department of Orthopedics, General Hospital of Southern Theater Command, Guangzhou, China; 3 Department of Hepatobiliary Surgery, The First Affiliated Hospital of Xi'an Jiaotong University, Xi'an, China; 4 Department of Hepatobiliary and Vascular Surgery, Ordnance Industry Health Research Institute (521 Hospital), Xi 'an, China; 5 Department of Medical Research, Key Laboratory of Environmental Sense Organ Stress and Health of the Ministry of Environmental Protection, The Ninth Medical Center of PLA General Hospital, Beijing, China; 6 Department of Aerospace Clinical Medicine, The Ninth Medical Center of PLA General Hospital, Beijing, China

**Keywords:** abdominal injury, animal model, blast injury, hepatic injury, novel hemostatic material

## Abstract

**Objective:**

To establish a stable porcine model of severe abdominal blast injury with massive hemorrhage and evaluate a novel hemostatic material (novel hydrogel bioadhesive) versus traditional gelatin for emergency management.

**Methods:**

A severe abdominal blast injury was induced in Bama miniature pigs (*n* = 18) using a 5 g JH-14 charge (3.5 cm standoff). The experimental animals were randomly divided into two cohorts: the blast injury model preparation cohort (*n* = 6) and the hemostatic material cohort (*n* = 12). The latter cohort was further randomized into control (gauze), hemostatic gelatin, and novel hemostatic material groups (*n* = 4/group). Hemostatic efficacy was assessed via vital signs, blood loss, survival, biomarkers, and histopathology.

**Results:**

The model produced full-thickness abdominal wall laceration (mean area: 16.24 ± 0.53 cm^2^), severe liver injury, and massive hemorrhage (497.8 ± 45.70 g blood loss), with systemic hypothermia and shock. Post-injury hematology showed reduced RBC, WBC, PLT, Hb, and ALB, while AST, ALT, and LDH surged. Both hemostatic materials reduced blood loss, extended survival, and partially stabilized vital signs, though Hb continued declining. Biochemical markers (ALT, LDH) partially recovered. Inflammatory markers (PCT, CRP, etc.) increased post-injury but remained unchanged post-treatment. Histopathology confirmed liver necrosis and inflammation, with partial recovery at 2 weeks; no apparent difference was observed between materials.

**Conclusion:**

The porcine model effectively simulates severe abdominal blast trauma. Both hemostatic materials achieved rapid local hemostasis but were limited by persistent occult bleeding and systemic complications. Future strategies should integrate optimized hemostasis with systemic resuscitation to enhance emergency outcomes.

## 1 Introduction

Under today’s complex and ever-changing environment, with the continuous advancement of technology, the threat of explosions from artillery shells, landmines, grenades, and other munitions in military combat scenarios, as well as the risk of explosion accidents in civilian domains such as industrial production, has been increasingly prevalent (1). A study by three Ukrainian researchers analyzing injury patterns among casualties during the Russia-Ukraine conflict (February to April 2022), compared to data from 2014 to 2021, revealed a 6% decrease in gunshot wounds and a 7% increase in blast injuries during the conflict (2). Another analysis of causes of soldier fatalities in international military operations also indicated that the majority of combat deaths are attributable to explosions (3). Research demonstrates that timely and appropriate first aid on the battlefield could save 15–25% of casualties who would otherwise die at the scene, with massive hemorrhage accounting for 90.9% of preventable combat-related deaths (4). Consequently, effective hemostasis during the early treatment phase (from the occurrence of combat trauma to transportation to a professional medical institution) is critical for preventing avoidable fatalities and remains a focal point in trauma care.

The anatomical locations of abdominal organs are dense and their functions are crucial. They involve multiple systems in the human body and jointly maintain the core physiological functions of life. This means that abdominal blast injuries often lead to severe consequences (5–7). For instance, they may cause extensive tissue loss, be accompanied by high infection rates, result in damage and exposure of critical organs, or even trigger massive hemorrhage. Treating abdominal blast injuries in the complex battlefield environment is exceptionally challenging (8, 9). Given the grave outcomes of such injuries, their management is undoubtedly a critical issue demanding high priority in the field of medical research.

The development of hemostatic agents has gained significant momentum in the medical field. Current research encompasses a diverse array of hemostatic materials, including mineral-based agents such as zeolite, kaolin, and mesoporous silica; chitosan-based agents derived from natural polysaccharides in insects, crustaceans, and fungal cell walls (10); plant-derived agents like oxidized cellulose, alginate, and starch; biologically sourced agents such as gelatin-, fibrin-, albumin-, keratin-, self-assembling peptide-, hyaluronic acid-, mussel protein/bioinspired-, and dextran-based formulations; and synthetic agents including poly(lactic-co-glycolic acid), polyethylene glycol, polyurethane, hydroxyethyl methacrylate, cyanoacrylate, superelastic hemostatic materials, hyperbranched polymers, shape-memory polymers, and aerogels. Tranexamic acid also falls within the scope of hemostatic research (11). Despite marked reductions in mortality from extremity hemorrhage, controlling junctional and intracavitary bleeding remains a formidable challenge (12), underscoring the urgent need for novel hemostatic materials to address deep, non-compressible hemorrhage.

Studies indicate that chitosan enhances hemostasis by aggregating erythrocytes, stimulating platelets, and accelerating coagulation. Ma et al. (13) demonstrated that quaternized chitosan amplifies both hemostatic and antibacterial efficacy, protecting wounds from infection while improving clotting. Guo et al. (14) showed that hydrophobically modified chitosan inserts into cell membranes via strong hydrophobic interactions, promoting blood cell aggregation and enhancing bactericidal properties of cationic polymers. Additionally, Oh et al. (15) reported that pyrogallol-functionalized chitosan hydrogels improve wet tissue adhesion and accelerate blood cell coagulation. Calcium ions, commonly incorporated into hemostatic agents, expedite thrombin generation by facilitating prothrombin conversion (14). Chitosan-Schiff base hydrogels further exhibit robust tissue adhesion, rapid gelation, hemostatic potency, and antimicrobial effects (16). Building on these advances, Zhao et al. (17) developed an injectable, antibacterial, self-expanding, self-propelling hydrogel bioadhesive by crosslinking lauric acid- and gallic acid-modified quaternized chitosan with oxidized dextran, integrating a foaming reaction between porous calcium carbonate and acetic acid. Traditional hemostatic methods have proven inadequate in managing massive intra-abdominal hemorrhage caused by blast injuries (18). In treating severe abdominal blast injuries with hemorrhage, novel hemostatic materials demonstrate significant potential, making their research an essential developmental trend (19–21). While this novel material effectively controlled non-compressible hemorrhage in porcine subclavian artery and vein transections, its efficacy against life-threatening hemorrhage from severe abdominal blast injuries remains unexplored. Similarly, hemostatic gelatin—a clinical mainstay for rapid hemostasis in minor vascular injuries—has not been validated for blast-induced massive hemorrhage.

Current studies on their application in such complex trauma scenarios remain extremely limited. There is not only a lack of systematic exploration into the efficacy of different hemostatic materials under these critical conditions but also insufficient understanding of their biocompatibility and safety within the intricate abdominal environment.

Due to the difficulty in replicating real-world blast scenarios and the poor reproducibility and stability of blast injury conditions, there is currently no ideal animal blast injury model for evaluating on-site emergency treatment efficacy. This study aims to establish a stable, reliable, and reproducible animal model of severe abdominal blast injury with massive hemorrhage using a miniature explosive device, facilitating research on damage control and field emergency care. Building on this model, we will further explore the preliminary effectiveness of novel hemostatic materials in emergency scenarios involving such injuries.

## 2 Materials and methods

### 2.1 Hemostatic materials and kits

The absorbable hemostatic gelatin matrix is a product based on porcine-derived gelatin, which has been approved for clinical use to facilitate the formation of blood clots and reduce intraoperative bleeding. (Surgiflo MS0010, Ferrosan Medical Devices A/S, Denmark) The novel hemostatic material was provided by the Institute of Frontier Science and Technology, Xi’an Jiaotong University. (Xi’an, China). The ELISA kits for Procalcitonin (PCT) (MK0071PA), Interleukin-1 (IL-1) (MK5148A), Interleukin-6 (IL-6) (MK2558A), Interleukin-10 (IL-10) (MK2565A), C-reactive protein (CRP) (MK2607A), and Tumor necrosis factor-alpha (TNF-α) (MK2523A) were all purchased from Jiangsu Addison Biotechnology Co., Ltd. (Yancheng, China). Traditional hemostatic gelatin is a sterile, absorbable porcine-derived gelatin fluid matrix. When applied to wounds, it rapidly promotes blood coagulation, reduces blood flow, and enhances vascular stability by forming an adherent film over the bleeding site. This mechanism facilitates immediate hemostasis. It is clinically indicated for controlling hemorrhage from capillaries, venules, and small arterioles when conventional methods (compression, ligation, etc.) prove ineffective or impractical. The novel hemostatic material employs covalent cross-linking of functionally modified chitosans to accelerate coagulation through enhanced conversion of prothrombin to thrombin. Concurrently, it exhibits superior tissue-adhesive properties and self-expanding capabilities, synergistically enabling rapid hemostasis.

### 2.2 Animals and experimental design

This study utilized healthy adult Bama miniature pigs (*Sus scrofa* domestica) to establish a severe abdominal blast injury animal model. Eighteen Bama miniature pigs, purchased from Xi’an Dilap Biomedical Co., Ltd. (License No. SCXK (Su) 2024-0012), regardless of sex, aged 8–10 months, and weighing 20–30 kg, were included. Standardized husbandry protocols were followed: the animal facility maintained a temperature of 18–25 °C, humidity of 60–70%, regular ventilation, and 12–14 h of daily light. Feed was provided according to growth stages with timed feeding, and water was available ad libitum. Daily cleaning and twice-weekly disinfection were performed by caretakers, and all animals underwent an acclimatization period of at least 7 days prior to experiments.

A total of 18 experimental animals were randomly assigned to two cohorts. The blast injury model preparation cohort (*n* = 6) was mainly to verify the validity and stability of the blast injury model preparation (see Section 2.3 for operational details and device setup), and the hemostatic material cohort (*n* = 12) was mainly used to evaluate the preliminary hemostatic effect of the hemostatic materials on the basis of the successful preparation of the blast injury model. As this was an exploratory pilot study, no formal sample size or power calculation was performed. The sample size of four animals per group was determined primarily on the basis of experimental feasibility and the ethical principles of the 3Rs. Animals were randomly allocated into three groups using a computer-generated randomization sequence, with stratification by body weight. Stratification was performed to ensure balance in body weight across groups. The randomization sequence was generated by independent personnel who were not involved in surgery or outcome assessment. Outcome assessors were blinded to group allocation.

*Control group*: Hemostasis was attempted solely with sterile gauze post-injury.

*Hemostatic gelatin group*: Hemostatic gelatin was applied for hemorrhage control.

*Novel hemostatic material group*: The novel hemostatic material was administered.

*Procedure*: After blast-induced injury, the abdominal cavity was immediately opened to rapidly and precisely identify the bleeding site. The designated hemostatic material was applied according to group assignment. In the gelatin group, two tubes of gelatin-based hemostatic material (approximately 16 mL in total) were injected or packed into the hemorrhagic area. In the novel hemostatic material group, one circular sheet of the novel hemostatic material (10.5 cm in diameter) was packed over the bleeding site. The amount of hemostatic material was standardized per animal. Following material application, sterile gauze was tightly packed over the wound to cover both the primary bleeding site and surrounding oozing regions, with moderate pressure maintained. The abdomen was subsequently wrapped with medical bandages to stabilize the gauze and prevent displacement. After hemostasis and bandaging were completed, fluid resuscitation was initiated via the auricular vein with 0.9% sodium chloride solution at 5–10 mL/kg/h and continued until transfer to a specialized medical facility, with a duration of approximately 1 h. The total infused volume was approximately 100–300 mL. The same fluid resuscitation protocol was used in all groups. All experimental protocols adhered to the guidelines of the Biomedical Ethics Committee of Xi’an Jiaotong University (Approval No. XJTUAE2024-1963) and complied with animal welfare standards.

### 2.3 Blast injury device and procedure

The explosive source consisted of a cylindrical JH-14 charge (density: 1.66 g/cm^3^, detonation velocity: 8,159 m/s, detonation pressure: 29.08 GPa) with a 5 g payload, initiated by an 8# electric detonator at the center of its end face. The blast device comprised two main components: a mobile animal restraint frame (for securing the experimental subject and explosive) and a measurement system. During experiments, the pig was positioned in lateral recumbency, with the charge fixed 3.5 cm from the liver’s surface. Free-field pressure sensors (PCB137B23, Hong Kong Champion International Semiconductor Limited, China) were deployed at horizontal distances of 29 cm (Sensor 1), 50 cm (Sensor 2), 100 cm (Sensor 3), and 120 cm (Sensor 4) from the charge center. These sensors were connected to a data acquisition system (GENESIS 7 t, Hottinger Brüel & Kjær, Germany) to record blast overpressure values.

The experimental animals were anesthetized with Zoletil 50, which contains 25 mg/mL tiletamine and 25 mg/mL zolazepam (total concentration, 50 mg/mL). The induction dose was 0.1 mL/kg intramuscularly, corresponding to 5 mg/kg total Zoletil. After blast injury, anesthesia was maintained with intermittent intravenous supplemental boluses of Zoletil at one-third to one-half of the induction dose, administered slowly as needed based on anesthetic depth, which was continuously monitored by the pedal withdrawal reflex (toe pinch), corneal reflex, heart rate, and arterial blood pressure. Analgesia was provided with butorphanol at 0.1–0.2 mg/kg intramuscularly. The liver was then ultrasonographically mapped using an ACUSON X150 (Siemens, Germany) to mark the blast target area. The animal was secured in lateral recumbency on the blast platform, and the explosive height was calibrated with vernier calipers before stabilizing the charge via a custom paper-cup fixture. Free-field sensors were positioned and connected to the acquisition system. After confirming circuit integrity, the detonator was connected, and the charge was detonated following safety verification. Post-detonation, data were collected in real time, and liver injury severity was immediately assessed. Throughout the procedure, spontaneous respiration was maintained, and multi-stage quality control measures ensured experimental reproducibility. Predetermined humane endpoints included refractory hypotension unresponsive to treatment, uncontrollable hemorrhage, persistent hypoxemia, refractory malignant arrhythmia, or uncontrolled severe pain. Animals meeting any of these criteria were euthanized immediately. At the end of the observation period or upon reaching a humane endpoint, euthanasia was performed by intravenous injection of sodium pentobarbital (≥100 mg/kg). Death was confirmed by the absence of spontaneous respiration, loss of pulse, and absence of corneal reflex, with observation continued for 5 min.

### 2.4 General condition assessment

Following blast injury, abdominal trauma was assessed by observing the wound site. A reference scale was placed adjacent to the injury, and photographs were taken perpendicularly above the wound to document the injury extent. The damaged area was quantified using ImageJ software (National Institutes of Health, USA) based on pixel-calibrated measurements. Blood loss was measured by weighing pre-weighed sterile gauze. During the experiment, blood was collected with pre-weighed sterile gauze. After the animal was transferred to the medical facility, the wound was opened, and all residual blood clots in the abdominal cavity were removed and weighed together with the blood-soaked gauze. The difference between the pre- and post-collection weights was converted to blood loss. This value represents the total blood loss collected before further surgical intervention, including blood collected by gauze and intra-abdominal blood clots, but excluding new or ongoing bleeding after further surgical intervention. Animal survival time was recorded using a digital timer.

### 2.5 Basic vital signs monitoring

Vital signs of the experimental animals, including body temperature (T), respiratory rate (R), heart rate (HR), diastolic blood pressure (DBP), systolic blood pressure (SBP), and mean arterial pressure (MAP), were monitored and recorded using an animal vital signs monitor (COMEN ND2-V, Shenzhen Comen Medical Instruments Co., Ltd., China) at three time points: before blast injury (baseline), within 5 min after blast injury, and approximately 1 h after hemostatic material application.

### 2.6 Blood routine parameter testing

Venous blood samples were collected from the experimental pigs at three time points: before blast injury (baseline), within 5 min after blast injury, and approximately 1 h after hemostatic material application. Within 2 h of collection, the samples were analyzed using an automated hematology analyzer (XN-1000, Sysmex Corporation, Japan) to assess routine blood parameters, including red blood cells (RBC), white blood cells (WBC), platelets (PLT), and hemoglobin (Hb).

### 2.7 Serum biochemical parameter testing

Venous blood samples were collected from the experimental pigs at three time points: before blast injury (baseline), within 5 min after blast injury, and approximately 1 h after hemostatic material application. Within 2 h of collection, serum was separated using a centrifuge (5425R, Eppendorf, Germany) at 3,000 rpm for 10 min and stored at −80 °C. For analysis, serum biochemical parameters—including albumin (ALB), albumin-to-globulin ratio (A/G), aspartate aminotransferase (AST), alanine aminotransferase (ALT), lactate dehydrogenase (LDH), creatine kinase (CK), creatinine (Cr), and blood urea nitrogen (BUN)—were measured using a fully automated biochemical analyzer (Roche Cobas 8000, Roche Diagnostics, Switzerland).

### 2.8 Inflammatory biomarker testing

Quantitative analysis of inflammatory markers, including PCT, IL-1, IL-6, IL-10, CRP and TNF-α, was performed using ELISA kits (Jiangsu Addison Biotechnology Co., Ltd., Yancheng, China) according to the manufacturer’s protocols.

### 2.9 Histopathological examination

Tissues were collected from all animals immediately after death or euthanasia. Animals that did not survive to 2 weeks were sampled immediately after death, whereas animals that survived to 2 weeks were euthanized at 2 weeks and sampled immediately after euthanasia. Liver, lung, kidney, spleen, small intestine, and heart tissue samples were collected during necropsy and fixed in 4% paraformaldehyde (Lablead, Beijing, China). The fixed tissues were dehydrated using a fully automated tissue dehydrator (JT-12S, Wuhan Junjie Electronics Co., Ltd., China), embedded in paraffin, and sectioned. Sections underwent dewaxing, hematoxylin staining, differentiation with hydrochloric acid-ethanol, bluing, eosin staining, and graded ethanol dehydration before being mounted with neutral resin. Pathomorphological changes were observed under a microscope (SQS-600P, Shenzhen Shengqiang Technology Co., Ltd., China).

### 2.10 Statistical analysis

Statistical analysis and graphical representation were performed using GraphPad Prism 9.5.0 (GraphPad Software, USA). Data are expressed as mean ± SD. Data distribution was assessed using the Shapiro–Wilk test, and homogeneity of variance was assessed using Levene’s test. Comparisons between two groups were performed using paired t-tests. Multi-group comparisons of single-factor data were performed using one-way ANOVA, followed by Tukey’s HSD *post-hoc* test. Two-factor data were analyzed using two-way repeated-measures ANOVA. Mauchly’s test was used to assess sphericity, and the Greenhouse–Geisser correction was applied when sphericity was violated. *Post-hoc* comparisons were adjusted using Bonferroni correction. Survival time was analyzed using restricted mean survival time (RMST), with a prespecified time point of *τ* = 3 h; observations beyond *τ* were censored at *τ*. Between-group comparisons were performed using permutation tests, and pairwise comparisons were adjusted using Holm correction. Significance levels are denoted as follows: ****p < 0.0001, ***p < 0.001, **p < 0.01, *p < 0.05.

## 3 Results

### 3.1 Blast overpressure

The experiment utilized a 5 g JH-14 charge composed of 96.5% RDX (cyclotrimethylenetrinitramine) and 3.5% additives, equivalent to approximately 1.25 times the TNT explosive yield. Blast overpressure curves at typical standoff distances (29 cm, 50 cm, 100 cm, and 120 cm) are shown in Figure 1. The overpressure values at each measurement point were calculated using a standard blast wave estimation formula (Equation 1), with results summarized in Table 1.

$$\mathrm{\Delta P}=\frac{0.084}{\bar{R}}+\frac{0.27}{{\bar{R}}^{2}}+\frac{0.7}{{\bar{R}}^{3}}$$ (1)

$$\bar{R}=\frac{\omega }{R}$$ (2)

**Figure 1:**
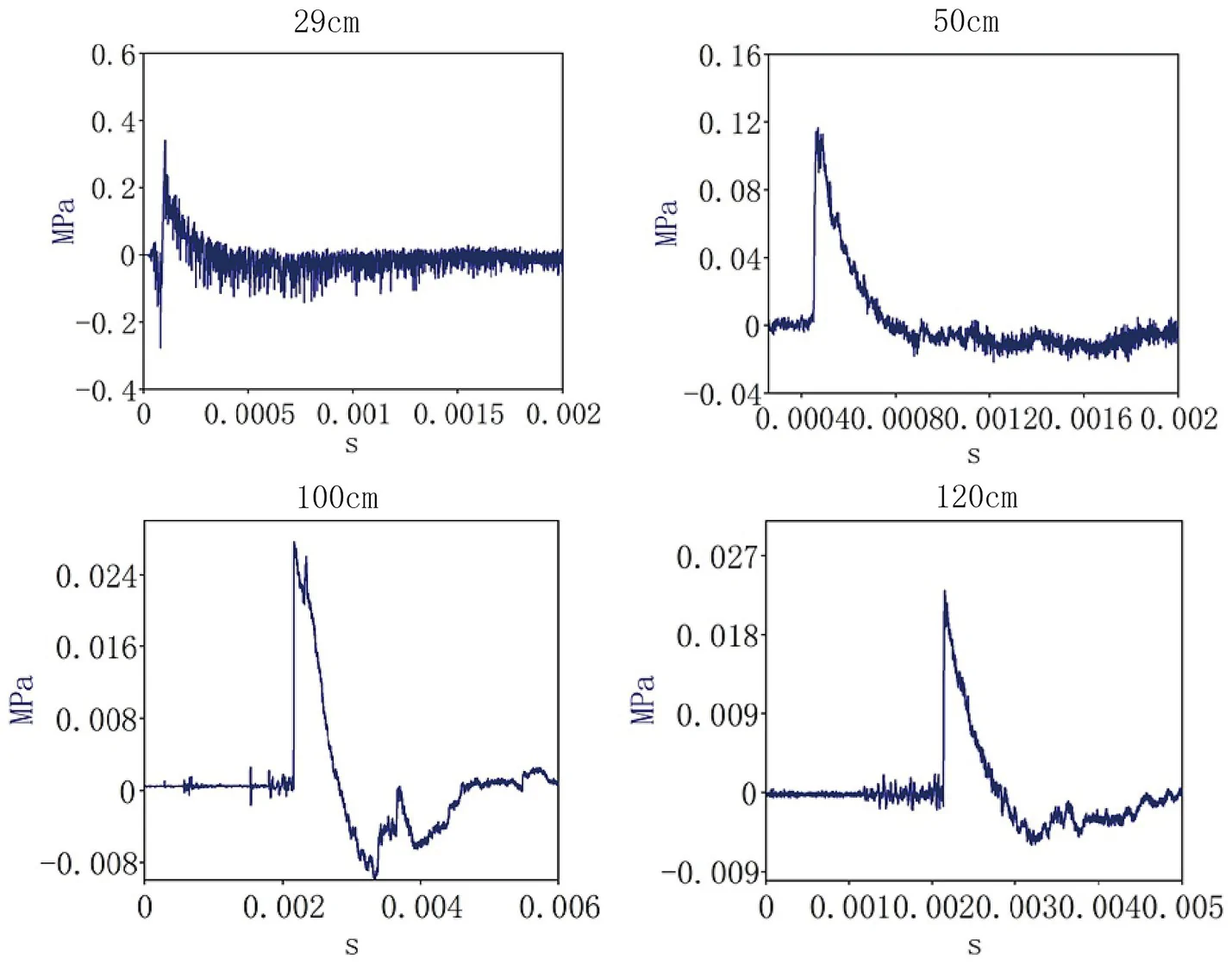
Blast overpressure curves at different standoff distances.

**Table 1:** Theoretical overpressure values at measurement points.

Standoff distance	0.29 m	0.50 m	1.00 m	1.20 m
Theoretical overpressure (MPa)	0.3417	0.1026	0.0290	0.0218
Experimental mean overpressure (MPa)	0.3095 ± 0.0255	0.117 ± 0.0127	0.0301 ± 0.0026	0.0222 ± 0.0018
Relative error (%)	10.4 ± 2.67	12.3 ± 1.34	3.6 ± 0.32	1.9 ± 0.15

In this context, ΔP represents the pressure variation (MPa) induced by the shock wave, *ω* denotes the equivalent TNT mass (kg), and R signifies the standoff distance (m) in experimental blast configurations (Equation 2). The equivalent TNT mass was fixed at 0.00625 kg according to experimental parameters. This formulation characterizes shockwave pressure attenuation by decomposing ΔP into the sum of three terms，each inversely proportional to the first, second, and third powers of scaled distance. It captures the progressive pressure decay during wave propagation, where distinct power terms dominate attenuation mechanisms at varying distance regimes. Such formulations are routinely applied in blast wave pressure field analysis and related research domains.

The theoretical estimates were compared against the average experimental data, as shown in Table 1. As evidenced by the experimental data, the measured blast wave parameters post-detonation show good agreement with theoretical values, confirming the applicability of theoretical formulas for computational purposes.

### 3.2 General post-blast injury characteristics

Following blast exposure, the pigs exhibited circular abdominal wall defects with full-thickness tissue destruction, fascial layer separation, hemorrhagic oozing from the wound surface, and charred appearance of the skin margins and small blood vessels. Upon opening the peritoneum, severe hepatic injury and significant intra-abdominal hemorrhage were observed. Statistical analysis of post-blast abdominal wall defect area and blood loss (Figure 2) revealed a mean wound area of 16.24 ± 0.53 cm^2^ (coefficient of variation, CV = 3.28%; <10%, indicating high consistency) and a mean blood loss of 497.8 ± 45.70 g (CV = 9.18%; <10%, highly consistent). These results demonstrate that a 5 g JH-14 charge with a standoff distance of 3.5 cm reliably produces abdominal injuries of uniform size and stable hemorrhage volume in the porcine model.

**Figure 2:**
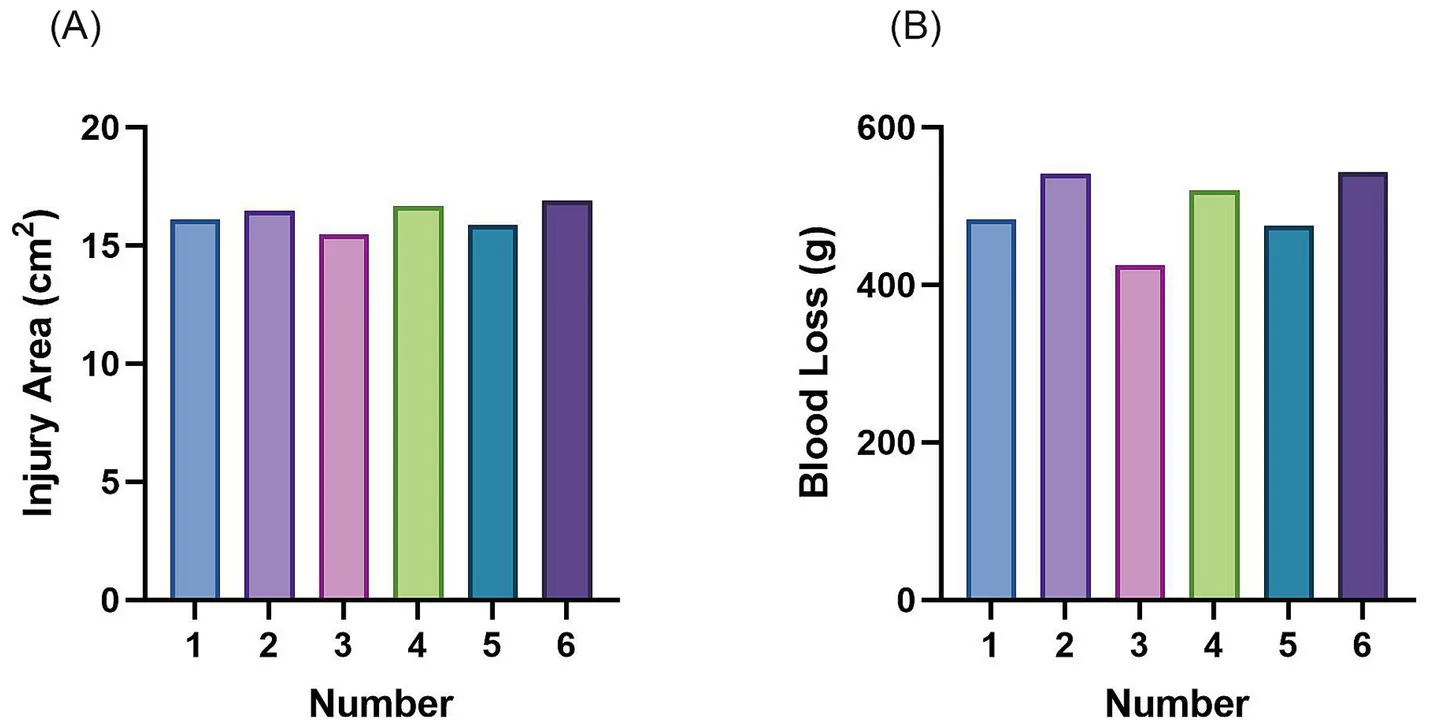
Abdominal wall defect area and blood loss following blast injury (*n* = 6). **(A)** Surface wound area in experimental animals post-blast; **(B)** blood loss in experimental animals post-blast injury.

### 3.3 Changes in vital signs following blast injury

Vital signs, including body temperature, respiratory rate, heart rate, systolic blood pressure, diastolic blood pressure, and mean arterial pressure, were measured and analyzed pre- and post-blast (Figure 3). Compared to pre-blast values, post-blast pigs exhibited significant decreases in body temperature and blood pressure (*p* < 0.01) and a significant increase in heart rate (*p* < 0.01). No statistically significant difference was observed in respiratory rate between pre- and post-blast conditions (*p* > 0.05).

**Figure 3:**
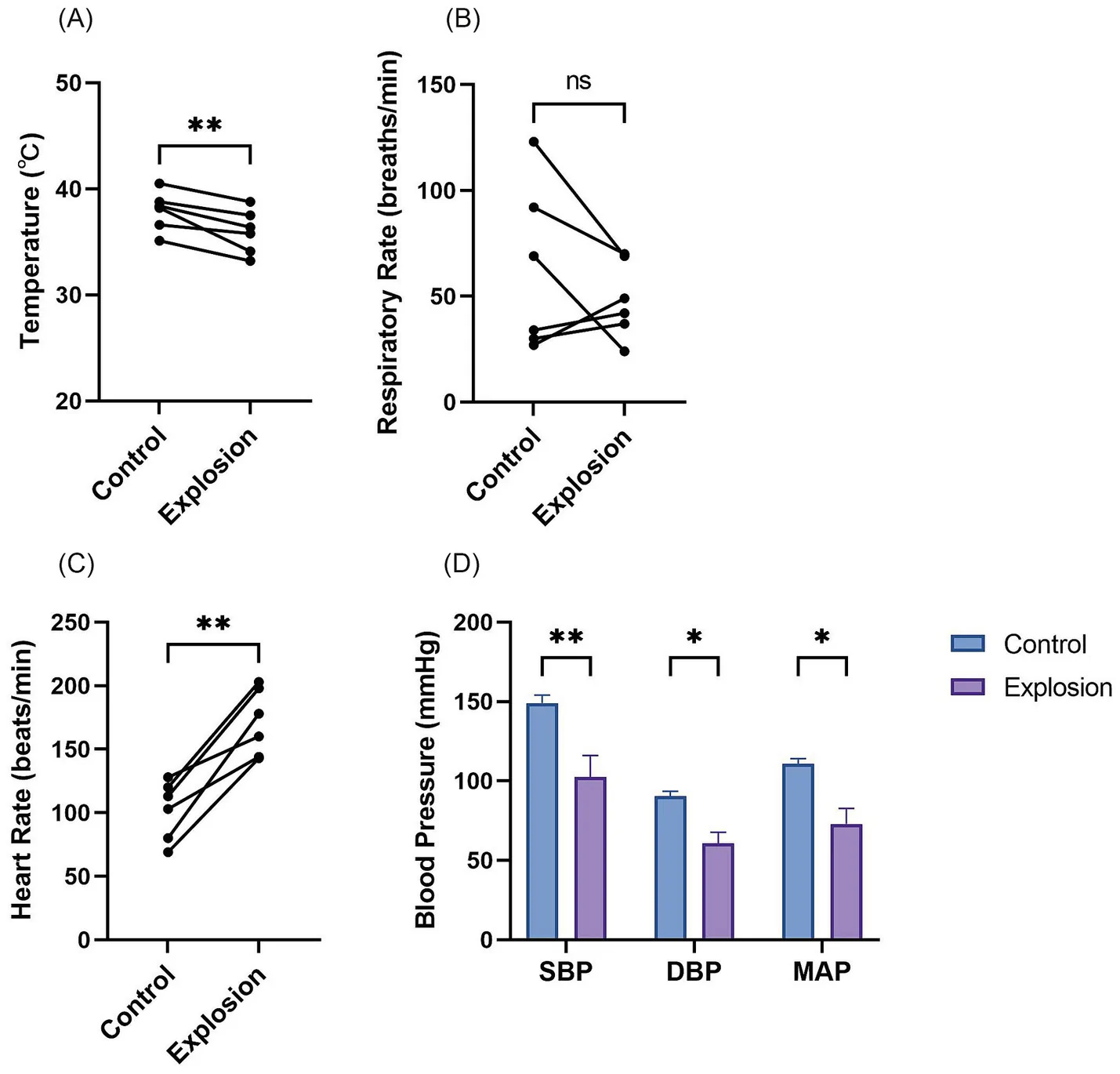
Changes in vital signs pre- and post-blast (*n* = 6). **(A)** Body temperature; **(B)** respiratory rate; **(C)** heart rate; **(D)** blood pressure changes pre- and post-blast injury *p < 0.05; **p < 0.01.

### 3.4 Changes in hematological and biochemical parameters post-blast injury

Following blast injury, statistically significant decreases (*p* < 0.05) were observed in porcine RBC, WBC, PLT, and Hb levels. Notably, RBC and PLT levels were significantly reduced (*p* < 0.01). Analysis of selected biochemical parameters pre- and post-blast (Figure 4) revealed a marked decline in ALB levels (*p* < 0.01) and elevated levels of AST, ALT, and LDH (*p* < 0.05). Among these, AST levels showed a pronounced increase (*p* < 0.01).

**Figure 4:**
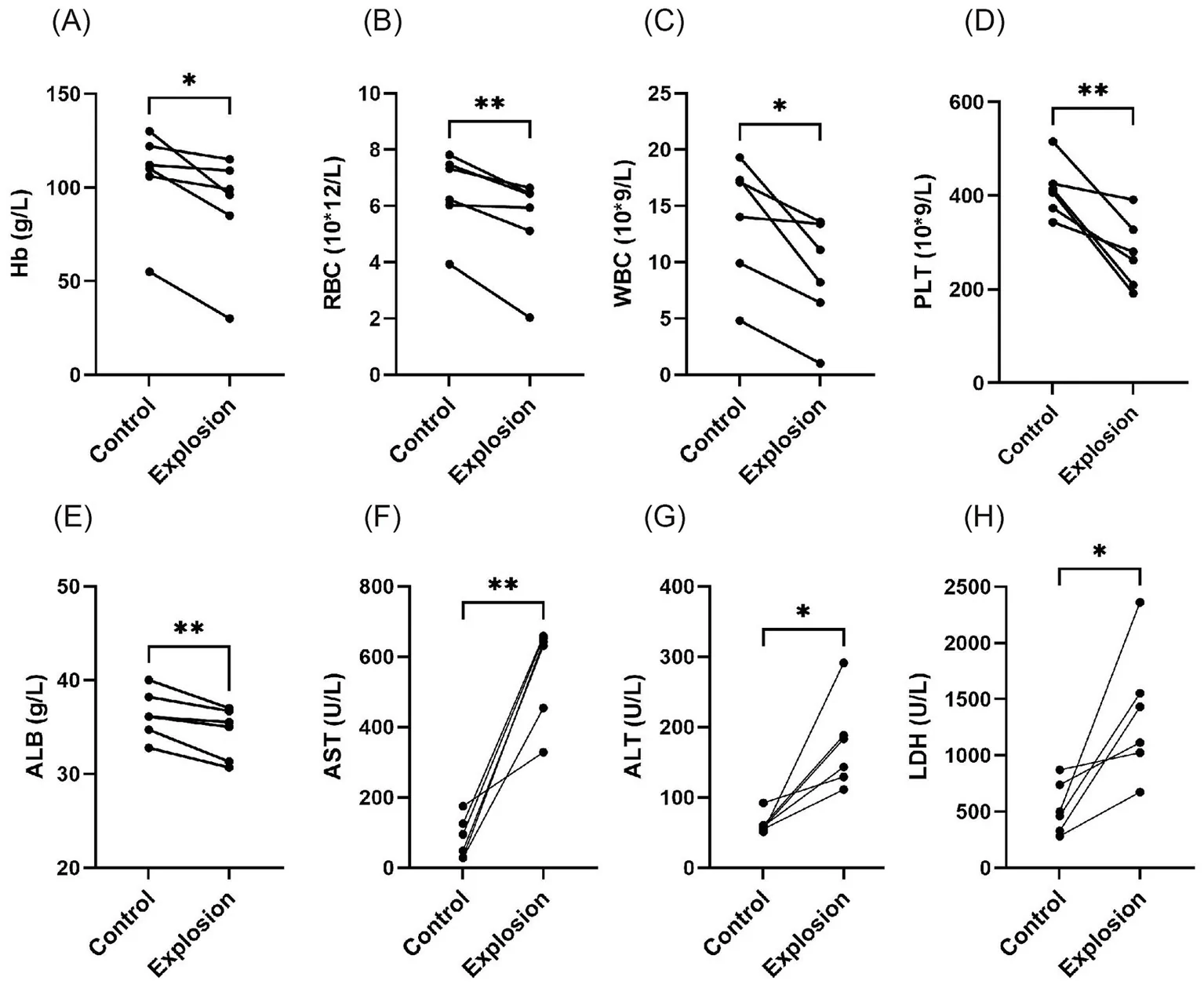
Changes in hematological and biochemical parameters pre- and post-blast (*n* = 6). **(A)** Hemoglobin; **(B)** red blood cells; **(C)** white blood cells; **(D)** platelets; **(E)** albumin; **(F)** aspartate aminotransferase; **(G)** alanine aminotransferase; **(H)** lactate dehydrogenase; *p < 0.05; **p < 0.01.

### 3.5 Gross and histopathological findings in liver post-blast injury

Following blast exposure, hepatic tissues exhibited marked blast wave-induced damage, characterized by radial lacerations, hemorrhage on the liver surface, and fragmentation of hepatic lobes. Histopathological analysis revealed focal hepatocyte degeneration and necrosis, with necrotic cells displaying cytoplasmic condensation or dissolution, nuclear pyknosis, and fragmentation. Progenitor cells surrounding necrotic areas were disorganized. Localized sinusoidal congestion, dilatation, and hemorrhage were observed, accompanied by scattered erythrocytes (Figure 5).

**Figure 5:**
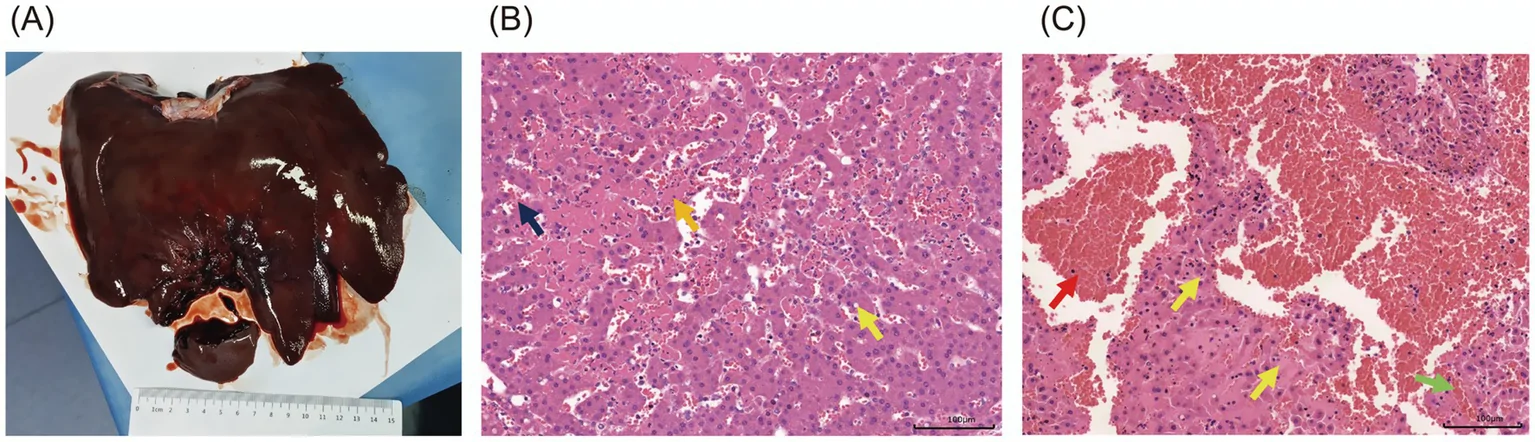
Gross and histopathological changes in porcine liver tissue post-blast injury (HE ×200). **(A)** Liver Injury Post-Blast; **(B)** necrotic hepatocytes (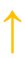) hepatocyte degeneration (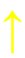) sinusoidal congestion and dilation (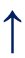); **(C)** hepatocyte degeneration and necrosis (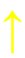) sinusoidal congestion and dilation (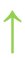) hemorrhage (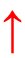).

### 3.6 General outcomes following hemostatic material application

After blast injury, animals exhibited standard blast injury characteristics, including irregular or circular abdominal defects with full-thickness abdominal wall damage, fascial layer separation, and hemorrhagic oozing from the wound surface. The animals were randomly divided into three groups for emergency intervention: sterile gauze (control group), hemostatic gelatin, and a novel hemostatic material. Survival time and blood loss were recorded and statistically analyzed across groups (Figure 6). Compared to the control group, both the hemostatic gelatin and novel hemostatic material groups showed significantly prolonged survival times (*p* < 0.05). However, no statistically significant difference was observed between the two treatment groups. Similarly, blood loss was significantly reduced in the hemostatic gelatin and novel material groups compared to the control (*p* < 0.05), with no significant difference between the two intervention groups.

**Figure 6:**
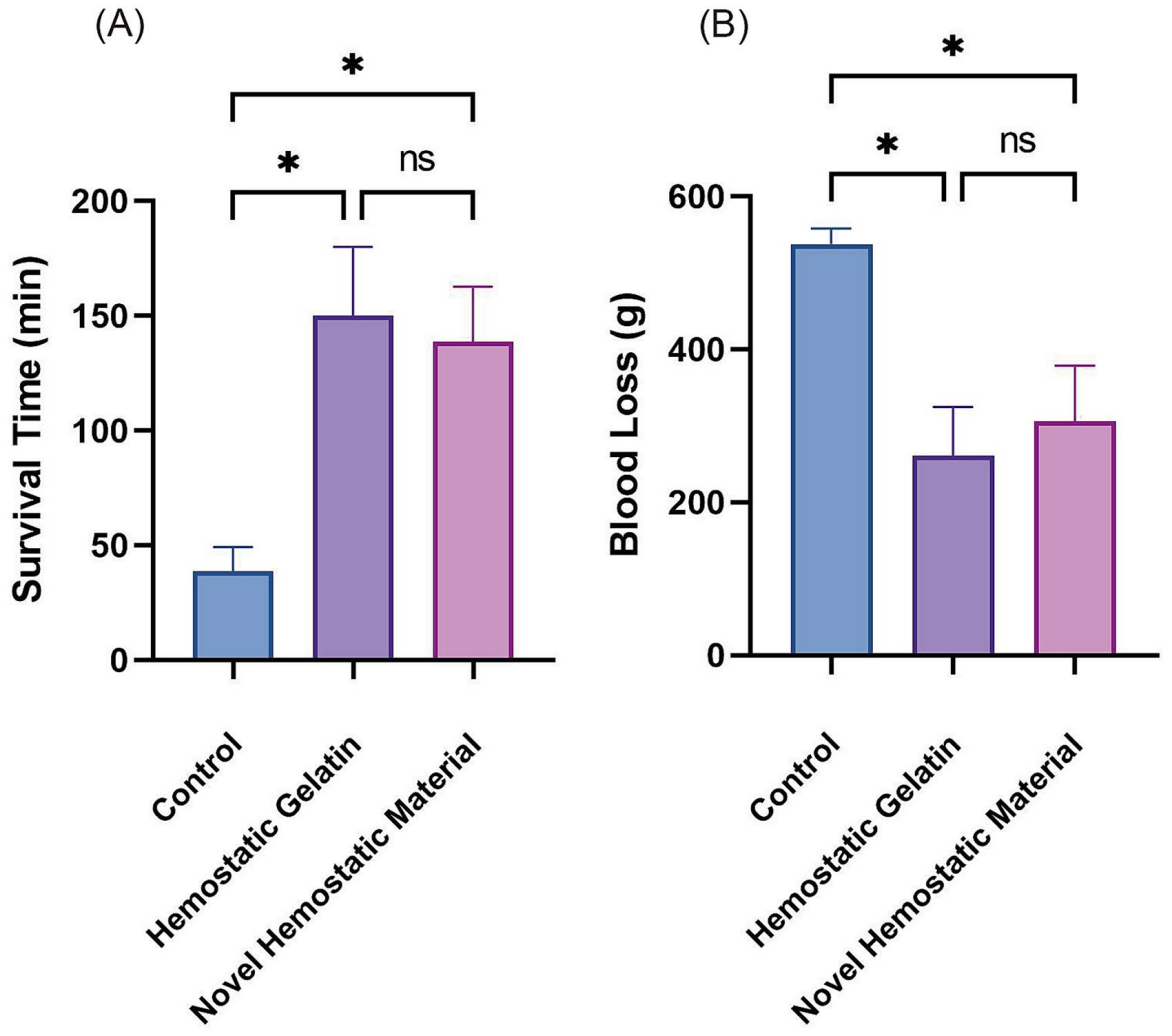
Analysis of hemostatic efficacy post-blast injury (*n* = 4). **(A)** Survival time; **(B)** blood loss *p < 0.05; ns, no significant difference.

### 3.7 Changes in vital signs following hemostatic material application

Vital signs, including body temperature, respiratory rate, heart rate, systolic blood pressure, diastolic blood pressure, and mean arterial pressure, were measured and analyzed pre-blast, post-blast, and post-treatment with different hemostatic materials (Figure 7). Post-blast injury, pigs exhibited a significant decrease in body temperature (*p* < 0.01). Following emergency intervention with hemostatic gelatin or the novel hemostatic material, body temperature partially recovered (*p* < 0.05), with no significant difference between the two materials (*p* > 0.05). Respiratory rate showed no consistent pattern across pre-blast, post-blast, and post-treatment phases. Compared to pre-blast values, heart rate increased markedly post-blast (*p* < 0.0001). Both hemostatic gelatin and the novel material reduced heart rate post-treatment (*p* < 0.05), though no significant difference was observed between the two interventions. Post-blast injury, blood pressure parameters (SBP, DBP, MAP) significantly declined (*p* < 0.01). Application of either hemostatic gelatin or the novel material effectively restored blood pressure to statistically significant levels (*p* < 0.05), with no intergroup differences.

**Figure 7:**
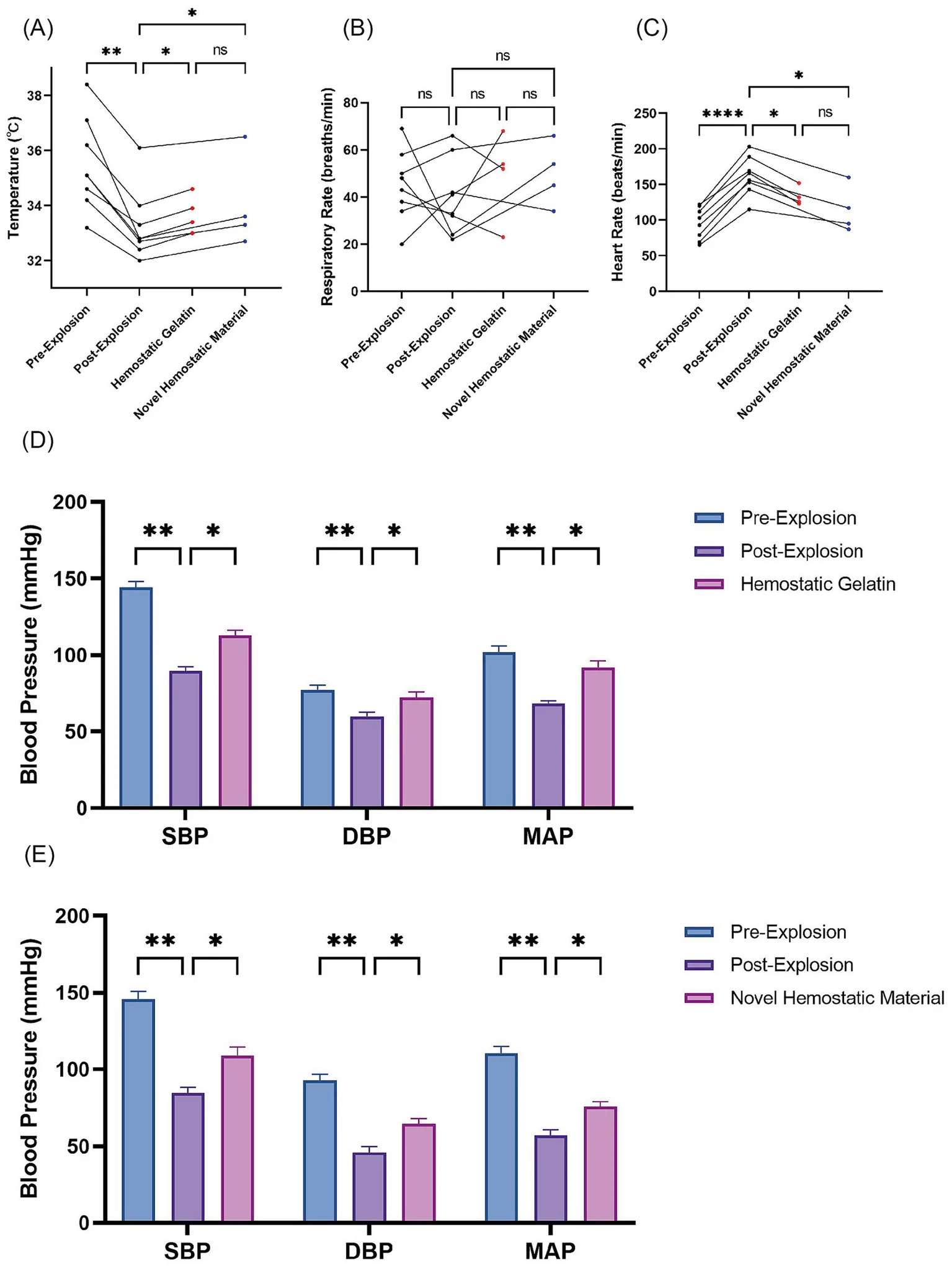
Changes in vital signs following application of different hemostatic materials (*n* = 4). **(A)** Body temperature; **(B)** respiratory rate; **(C)** heart rate; **(D)** blood pressure changes post-hemostatic gelatin treatment; **(E)** blood pressure changes post-novel hemostatic material treatment; *p < 0.05; **p < 0.01; ****p < 0.0001; ns, no significant difference.

### 3.8 Changes in hematological parameters following hemostatic material application

Compared to pre-blast values, post-blast injury resulted in statistically significant reductions in RBC, WBC, PLTs, and Hb levels (*p* < 0.05), with RBC showing a pronounced decrease (*p* < 0.001). Treatment with the novel hemostatic material or hemostatic gelatin attenuated the decline in RBC, WB, and PLTs. Although Hb levels continued to decrease post-treatment, the differences were not statistically significant. Furthermore, no significant difference in therapeutic efficacy was observed between the novel material and hemostatic gelatin (Figure 8).

**Figure 8:**
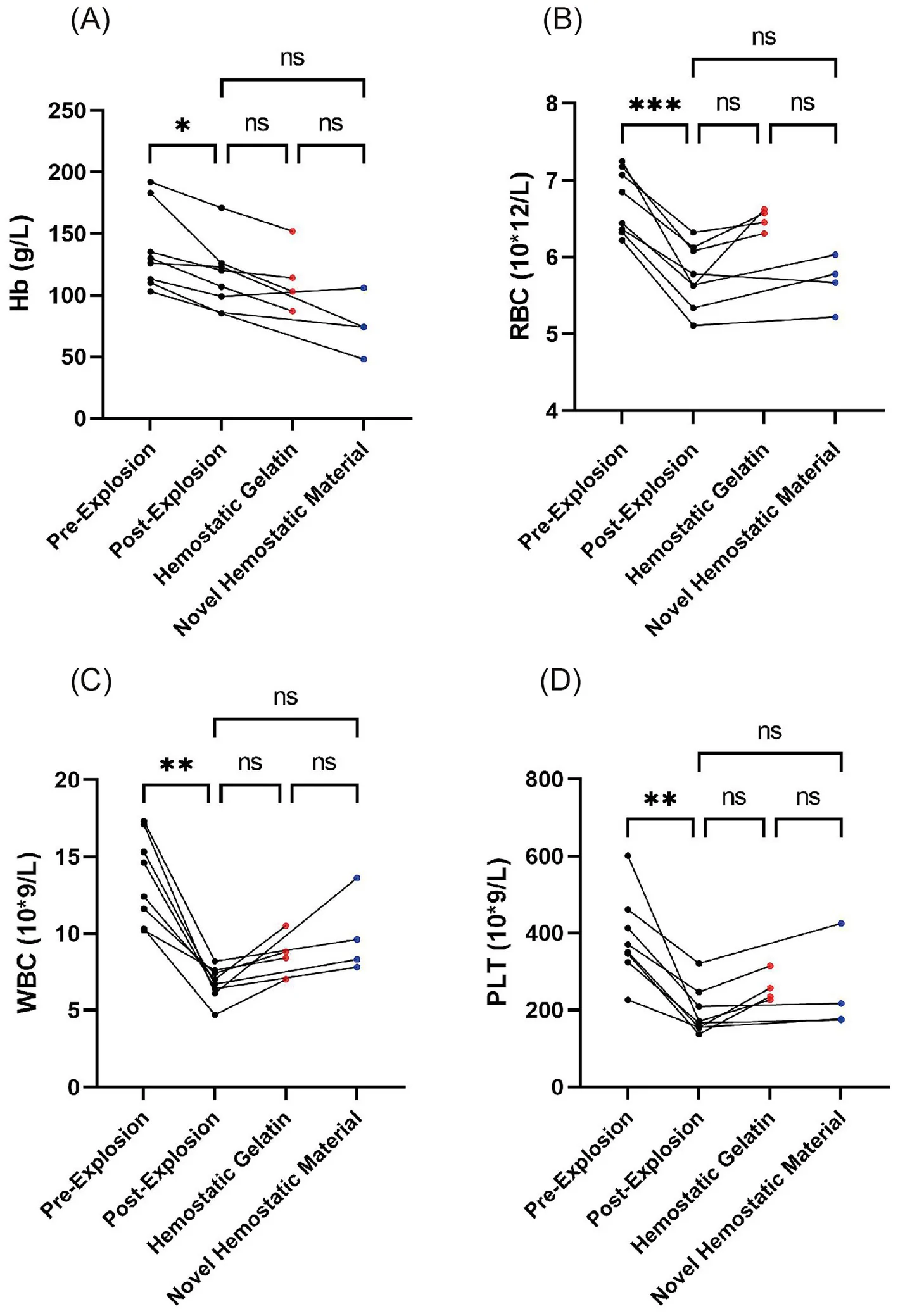
Changes in hematological parameters following application of different hemostatic materials (*n* = 4). **(A)** Hemoglobin; **(B)** red blood cells; **(C)** white blood cells; **(D)** platelets; *p < 0.05; **p < 0.01; ***p < 0.001; ns, no significant difference.

### 3.9 Changes in biochemical parameters following hemostatic material application

Analysis of biochemical parameters pre-blast, post-blast, and post-treatment with the novel hemostatic material or hemostatic gelatin (Figure 9) revealed significant post-bast reductions in ALB, A/G, and Cr levels (*p* < 0.05), alongside elevated levels of AST, ALT, LDH, CK and BUN (*p* < 0.05). Notably, AST, ALT, LDH, and CK levels increased markedly (*p* < 0.0001). Post-treatment, only ALT and LDH levels showed statistically significant recovery (*p* < 0.05), while other parameters remained unchanged or exhibited non-significant trends. No significant difference in therapeutic outcomes was observed between the novel material and hemostatic gelatin.

**Figure 9:**
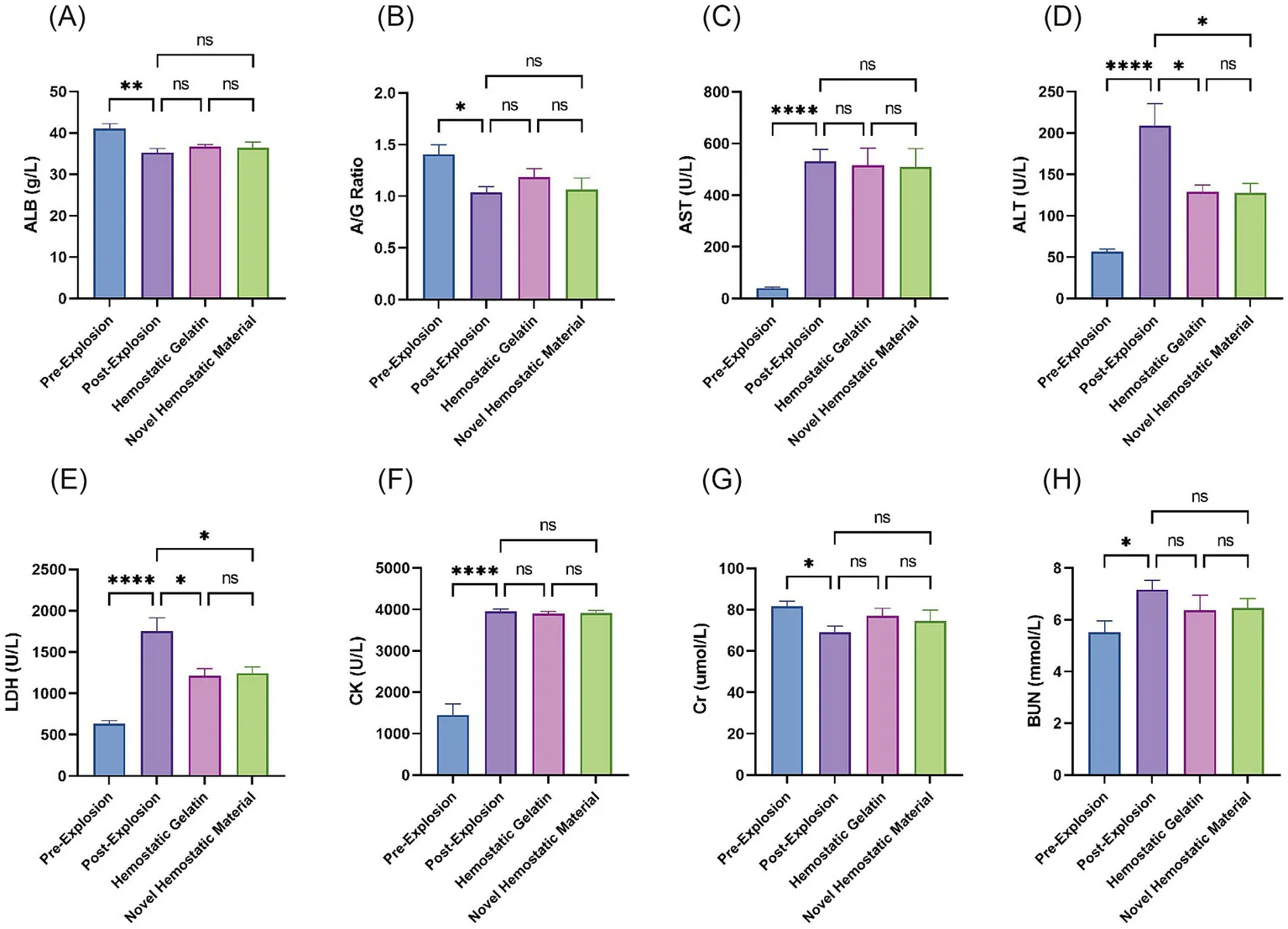
Changes in biochemical parameters following application of different hemostatic materials (*n* = 4). **(A)** Albumin; **(B)** albumin-to-globulin ratio; **(C)** aspartate aminotransferase; **(D)** alanine aminotransferase; **(E)** lactate dehydrogenase; **(F)** creatine kinase; **(G)** creatinine; **(H)** blood urea nitrogen; *p < 0.05; **p < 0.01; ****p < 0.0001; ns, no significant difference.

### 3.10 Changes in inflammatory markers following hemostatic material application

Changes in inflammatory markers, including PCT, CRP, TNF-α, IL-1, IL-6, and IL-10, were measured pre-blast, post-blast, and post-treatment with the novel hemostatic material or hemostatic gelatin using ELISA kits (Figure 10). Compared to pre-blast levels, all inflammatory markers significantly increased post-blast (*p* < 0.01). No statistically significant changes were observed in these biomarkers following treatment with either the novel material or hemostatic gelatin compared to post-blast values. Additionally, no significant difference in therapeutic efficacy was detected between the two materials.

**Figure 10:**
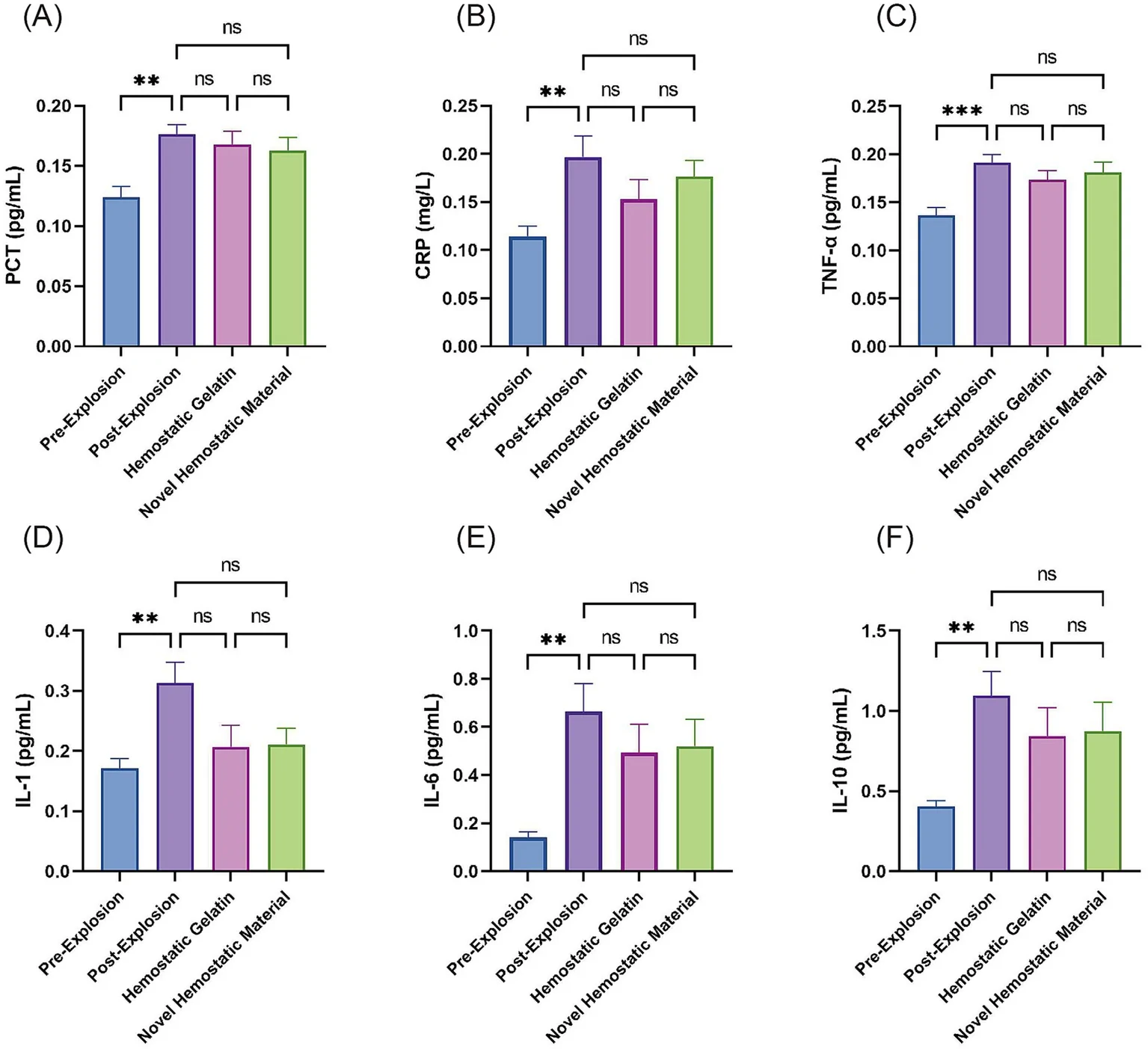
Changes in inflammatory markers following application of different hemostatic materials (*n* = 4). **(A)** Procalcitonin; **(B)** C-reactive protein; **(C)** tumor necrosis factor-alpha; **(D)** interleukin-1; **(E)** interleukin-6; **(F)** interleukin-10; **p < 0.01; ***p < 0.001; ns, no significant difference.

### 3.11 Histopathological changes following hemostatic material application

Post-bast injury, hepatic tissues exhibited extensive hepatocyte degeneration, necrosis, and inflammatory cell infiltration. After treatment with the novel hemostatic material or hemostatic gelatin, only minor hepatocyte degeneration and scattered lymphocytes were observed, with no significant difference between the two treatments. In lung tissues, blast injury induced necrosis, inflammatory cell infiltration, fibrotic hyperplasia, and fibrinoid exudation. Post-treatment, both materials showed residual inflammatory infiltration and fibrotic hyperplasia, with no apparent difference in histological findings between them (Figure 11). In renal tissue, cellular casts and inflammatory cell infiltration were observed after blast injury, both of which improved following treatment with hemostatic materials (Supplementary Figure S1). No apparent histopathological abnormalities were observed in the other tissues such as the heart, spleen, and small intestine (Supplementary Figure S2).

**Figure 11:**
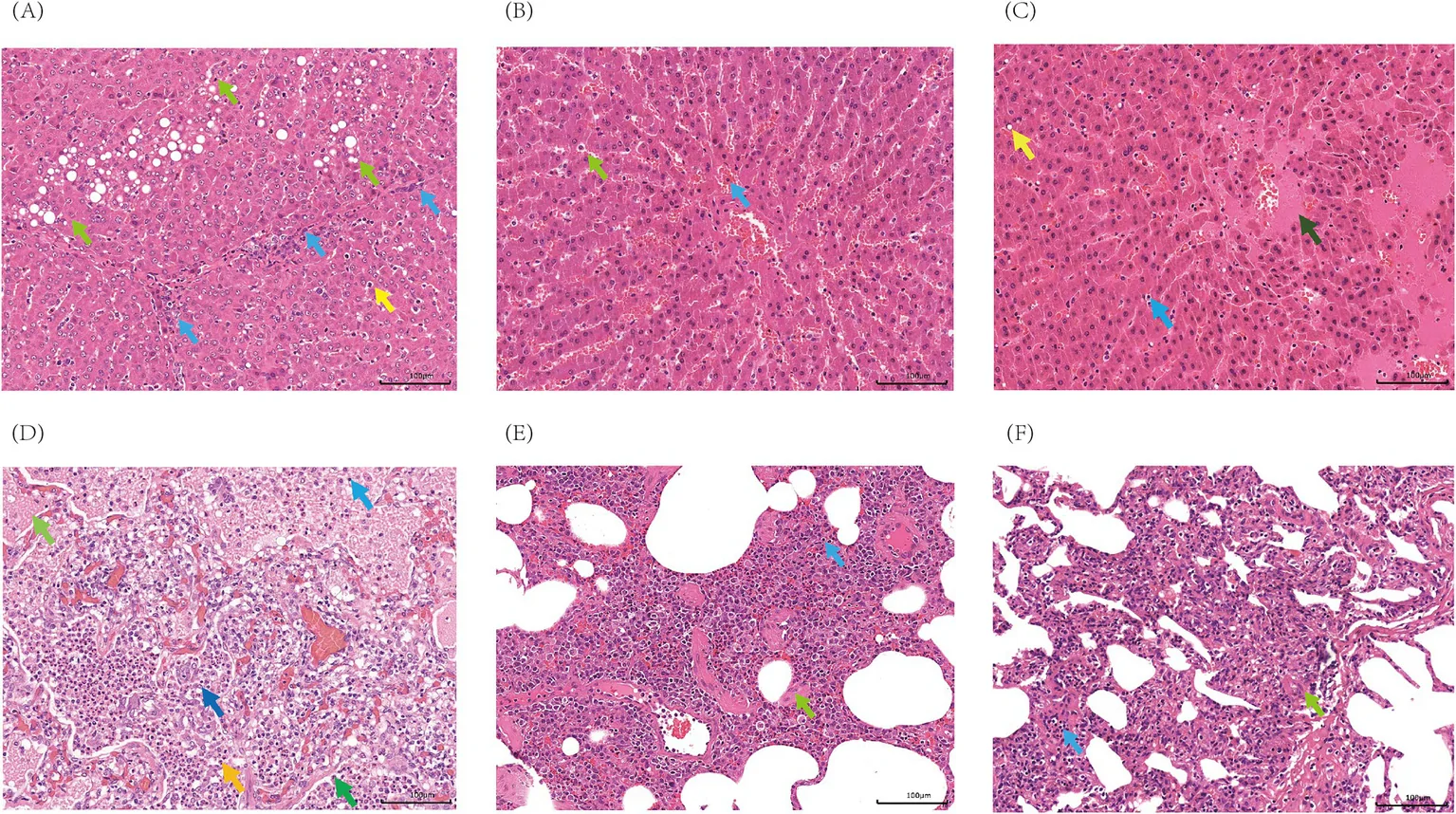
Histopathological changes in liver and lung tissues βfollowing application of different hemostatic materials (HE×200). **(A)** Liver post-blast injury: hepatocyte degeneration (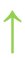) lymphocytes (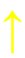) inflammatory cell infiltration (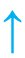); **(B)** liver 2 weeks post-hemostatic gelatin treatment: hepatocyte degeneration (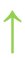) lymphocytes (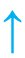); **(C)** liver 2 weeks post-novel hemostatic material treatment: hepatocyte degeneration (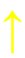) lymphocytes (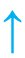) serous exudate (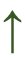); **(D)** lung post-blast injury: necrotic cellular debris (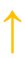) neutrophils (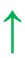) lymphocytes (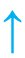) fibroblasts (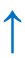) fibrinoid exudate (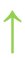); **(E)** lung 2 weeks post-hemostatic gelatin treatment: lymphocytes (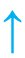) fibroblasts (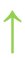); **(F)** lung 2 weeks post-novel hemostatic material treatment: lymphocytes (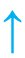) fibroblasts (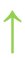).

## 4 Discussion

With the continuous advancement of technology and widespread use of advanced weaponry, global explosive-related injuries have surged. Neeman et al. (22) analyzed injury patterns during the “Iron Sword” operation in Gaza, revealing that explosions were the primary mechanism of combat-related trauma. Tiruneh et al. (23) compared casualty characteristics and outcomes between the 2023 and 2014 major conflicts between Israel and Hamas, demonstrating a significant increase in injury severity among soldiers in 2023, predominantly polytrauma, with abdominal injuries rising from 14.2 to 20.9%. The abdomen houses numerous visceral organs that are relatively fragile, and its thin abdominal wall provides limited protection against blast waves. Consequently, shockwaves generated during explosions readily induce internal organ rupture and hemorrhage. Although abdominal blast injuries are characterized by polytrauma, high severity, elevated infection rates, and challenging post-injury management, they exhibit greater salvageability compared to thoracic and craniocerebral blast injuries, making them an ideal model for studying emergency interventions.

An explosion is defined as the rapid conversion of solid or liquid substances into gaseous forms with instantaneous energy release (24). Explosives can be broadly classified into two categories based on detonation velocity and energy output: high-order explosives (generating supersonic detonation waves) and low-order explosives (producing subsonic deflagration). High-order explosives generate a “blast wave” characterized by a steep pressure front exceeding ambient atmospheric pressure by up to 30,000-fold, propagating at velocities reaching 8,000 m/s (17). As the blast wave travels, its energy dissipates and pressure attenuates. The subsequent blast wind—composed of high-velocity, superheated air—propels debris and collapses structures, causing blunt and penetrating injuries (18). Within the abdomen, shear stress arising from differential tissue motion (due to varying wave propagation speeds across organs of differing densities) leads to tissue laceration and organ displacement (25).

Lu et al. developed a severe hepatic trauma model using a self-designed *in vivo* blast injury device, where black powder was detonated on the surface of miniature pigs’ hepatic lobes via surgical laparotomy (26–28). While this model demonstrated good reproducibility and controllability, it faced limitations in standardizing explosive specifications (e.g., formulation, dosage) and ignored the protective role of the intact abdominal wall during blast exposure. Zhou et al. simulated fragment injuries by attaching TNT-coated steel beads to one side of explosives, but this approach neglected blast wave effects and resulted in complex trauma involving hollow viscera, parenchymal organs, and vasculature, complicating standardized emergency care research (29–31). Building on prior studies, our research introduced improvements: a 5 g JH-14 charge was employed as the explosive source, with pre-blast ultrasonic localization of the liver to standardize explosive parameters and better simulate real-world blast scenarios. The small charge mass and precise targeting minimized post-blast injury complexity, enhancing model stability and facilitating reproducible studies on emergency interventions.

To optimize the severity and reproducibility of blast injuries, preliminary experiments were conducted at varying standoff distances. When the explosive charge was positioned 1.5 cm from the body surface, it caused full-thickness abdominal wall lacerations, extensive hepatic surface injuries, and massive intra-abdominal hemorrhage, leading to rapid animal death (within 3 min) and rendering resuscitation impractical. In contrast, a standoff distance of 6.0 cm resulted in minor skin defects without full-thickness abdominal wall penetration, minimal hemorrhage, and long-term survival after basic wound closure, thereby lacking the urgency required for emergency care studies. At an intermediate distance of 4.5 cm, injuries included partial-thickness abdominal wall damage (sparing the peritoneum), hepatic contusions/lacerations, and significant intra-abdominal hemorrhage. Although the abdominal wall remained partially intact, the extent of visceral injury and hemorrhage met the criteria for severe trauma. Based on these findings, the final standoff distance was refined to 3.5 cm to further amplify injury severity, ensuring consistent establishment of a severe abdominal blast injury model with life-threatening hemorrhage while maintaining reproducibility for emergency intervention research.

Analysis of post-blast injury characteristics revealed that the JH-14 charge employed in this study induced severe abdominal blast injuries in experimental animals, including full-thickness abdominal wall lacerations and multi-organ damage (notably hepatic injury), accompanied by massive intra-abdominal hemorrhage. Without effective emergency intervention, animals succumbed rapidly, failing to survive long enough to reach specialized medical facilities—a scenario mirroring the critical “golden hour” concept [the 60-min window for delivering life-saving surgical care post-injury (32)]. The model of severe blast injury with hemorrhage established here reproduces the key features of blast-induced abdominal injury with massive hemorrhage and shows preliminary consistency in abdominal wall defect area and blood loss. It may serve as a useful tool for investigating this time-sensitive clinical challenge.

Regarding vital signs, all pigs exhibited consistent physiological trends post-blast: decreased body temperature, slowed respiration, elevated heart rate, and reduced blood pressure, indicative of varying degrees of hemorrhagic shock. These changes, except for respiratory rate, showed statistically significant differences compared to pre-blast baselines (*p* < 0.05), aligning with prior reports of blast-induced hypothermia, shock, and respiratory depression (33, 34). These findings confirm that the observed physiological alterations are consistent with established blast injury pathophysiology.

Analysis of hematological and biochemical parameters demonstrated that blast injury induced significant reductions in RBC, WBC, PLT, Hb and ALB, coupled with elevated levels of AST, ALT, and LDH. These alterations may involve multiple interrelated mechanisms (35, 36). Massive hemorrhage may reduce the effective circulating blood volume and lead to declines in RBC and Hb, although hemodilution caused by fluid resuscitation may also contribute. Changes in WBC counts may reflect acute stress and cell redistribution. PLT reduction may be related to platelet consumption or hemodilution after hemorrhage. Elevated AST and ALT directly reflected blast-induced hepatic injury; and increased LDH levels indicated widespread cellular necrosis and tissue damage, as intracellular enzymes were released into the bloodstream. Histopathological findings further confirmed hepatic pathology consistent with blast trauma, including hepatocyte degeneration/necrosis, inflammatory infiltration, serous exudation, sinusoidal congestion, and hemorrhage.

Collectively, the consistency of model preparation was primarily assessed based on the abdominal wall defect area and blood loss in the six animals of the model preparation cohort, showing preliminary consistency. In addition, post-blast vital signs, hematological parameters, biochemical indices, and histopathological findings were consistent with blast injury, providing supportive evidence for model validity. This establishes a robust platform for investigating injury mechanisms and evaluating emergency interventions in trauma scenarios.

The results demonstrated that both the novel hemostatic material and hemostatic gelatin significantly reduced blood loss and prolonged survival time in animals with severe abdominal blast injuries, indicating their efficacy in managing massive hemorrhage and extending the critical intervention window. However, substantial residual hemorrhage persisted, with survival times extending only to approximately 3 h, underscoring the partial nature of their hemostatic effects. Analysis of post-treatment vital signs revealed that both materials restored blood pressure and body temperature while lowering heart rate, though no significant difference in efficacy was observed between them. These findings suggest that both hemostatic materials may have some hemostatic effect. However, owing to the small sample size and limited statistical power, the present results cannot be interpreted as evidence that the two materials have comparable efficacy. Larger randomized, blinded, controlled studies are needed to further evaluate their efficacy in hemorrhage control and physiological stabilization. Hematological parameters showed transient recovery in RBC, WBC and PLT post-treatment, though Hb levels continued to decline. This may reflect ongoing occult intra-abdominal bleeding and/or hemodilution caused by fluid resuscitation. Notably, during the experiment, after reopening the abdominal wound, the hemostatic material was observed to cover the surface of the liver wound; however, because the wound area was large and irregularly shaped, complete and effective hemostasis was difficult to achieve, and oozing from the wound persisted. This suggests the possibility of ongoing bleeding from the wound. Therefore, the continued decline in Hb may have resulted from ongoing bleeding that exceeded the body’s capacity to replenish Hb, although a contribution from hemodilution cannot be excluded. These findings suggest that, in this severe trauma model, the hemostatic materials may not have fully controlled bleeding, and their efficacy requires further improvement and validation. Biochemical analysis revealed statistically significant changes only in ALT and LDH, likely due to the acute nature of hepatic injury and cellular necrosis. Other parameters, often reflective of longer-term metabolic shifts, remained unaffected, possibly owing to the localized application of hemostatic agents. Inflammatory markers, including PCT, CRP, TNF-α, IL-1, IL-6, and IL-10, were elevated after blast injury, suggesting a possible systemic inflammatory response to trauma. Neither material significantly modulated these biomarkers, potentially due to the complexity of inflammatory cascades and the delayed systemic effects of localized hemostatic interventions. Because most animals died within approximately 3 h after hemostatic intervention, histopathological evaluation at 2 weeks was limited to one animal per group in the two hemostatic material groups; no control animal survived to 2 weeks. Histopathological evaluation at 2 weeks post-treatment showed partial recovery of hepatic and pulmonary injuries compared to acute blast damage, with no apparent difference in reparative outcomes.

In conclusion, both the novel hemostatic material and hemostatic gelatin demonstrated comparable, yet incomplete, efficacy in controlling hemorrhage and prolonging survival in severe abdominal blast injuries. While they provide critical temporary stabilization, further innovation is urgently needed to develop more robust hemostatic strategies for field resuscitation in trauma scenarios.

The established severe abdominal blast injury model serves as a high-fidelity screening platform for evaluating the efficacy of hemostatic agents in field resuscitation scenarios. Future refinements will incorporate multimodal injury factors (e.g., thermal effects, fragmentation) to better simulate combat wound environments. Longitudinal studies tracking long-term complications—including infection, adhesion formation, and multi-organ dysfunction—combined with multi-omics approaches will elucidate underlying molecular mechanisms. For next-generation hemostat development, enhancing tissue adhesion robustness remains a critical focus.

## 5 Conclusion

This study demonstrates that a severe abdominal blast injury model in swine was successfully established using a 5 g JH-14 charge at a standoff distance of 3.5 cm. The model effectively simulates explosion-related abdominal injuries with massive hemorrhage, exhibiting stability, operability, and reproducibility, thereby providing a critical tool for investigating blast injury mechanisms and evaluating emergency interventions. Both the novel hemostatic material and conventional hemostatic gelatin demonstrated comparable efficacy in controlling intra-abdominal hemorrhage, reducing blood loss, and prolonging survival time, creating a vital window for casualty evacuation to rear medical facilities. However, the partial nature of this hemostatic effect underscores the urgent need to explore more robust materials and strategies to address life-threatening hemorrhage in prehospital care.

## 6 Advantages and limitations of the study

### 6.1 Advantages

In this study, a miniature explosion source was used to prepare an animal model of severe abdominal explosion injury, which is conducive to exploring the mechanism of explosion damage and providing a research platform for emergency treatment at the explosion site. In addition, the hemostatic effect of hemostatic materials can be evaluated and verified.

### 6.2 Limitations

Several limitations of this study should be acknowledged. First, the treatment groups were small, and the statistical power was limited. Larger randomized, blinded, controlled studies are needed to verify the comparative efficacy of the two materials. Second, because the control animals did not survive to the post-treatment time point, the post-treatment changes after hemostatic intervention reflect only the surviving animals and cannot be directly compared with the control group. Therefore, the potential contributions of time, fluid resuscitation, or supportive care to the observed results cannot be excluded, and these post-treatment changes should be considered exploratory findings that cannot be entirely attributed to the hemostatic materials. Future studies should consider adjusting the severity of the hemorrhage model or setting earlier blood sampling time points to obtain control group data and enable a more comprehensive between-group comparison. Third, the histopathological findings are exploratory and descriptive and require further validation in larger studies. Fourth, the hemostatic materials showed limited efficacy in this severe emergency model, which may be related to their shape, texture, method of application, and application scope. In addition, this study focused mainly on rapid hemostatic efficacy and paid limited attention to long-term recovery. Future studies could develop materials more suitable for rapid hemostasis in abdominal injuries and further evaluate their long-term effects.

## Data Availability

The original contributions presented in the study are included in the article/Supplementary material, further inquiries can be directed to the corresponding authors.

## Supplementary material

SUPPLEMENTARY FIGURE S1 Histopathological changes in kidney tissues following application of different hemostatic materials (HE×200). **(A)** Kidney post-blast injury: cellular cast () lymphocytes () neutrophils () fibroblasts (); **(B)** kidney 2 weeks post-hemostatic gelatin treatment: renal tubular epithelial cell degeneration () renal tubular dilation (); **(C)** kidney 2 weeks post-novel hemostatic material treatment: renal tubular epithelial cell degeneration ()

SUPPLEMENTARY FIGURE S2 Histopathological changes in heart, spleen and small intestine tissues following application of different hemostatic materials (HE×200). **(A)** Heart post-blast injury; **(B)** heart 2 weeks post-hemostatic gelatin treatment; **(C)** heart 2 weeks post-novel hemostatic material treatment; **(D)** Spleen post-blast injury; **(E)** spleen 2 weeks post-hemostatic gelatin treatment; **(F)** spleen 2 weeks post-novel hemostatic material treatment; **(G)** small intestine post-blast injury; **(H)** small intestine 2 weeks post-hemostatic gelatin treatment; **(I)** small intestine 2 weeks post-novel hemostatic material treatment.
